# Comparison of verbal autopsy using a large language model to biologically confirmed causes of death for malaria and other communicable diseases among children in six sub-Saharan African countries

**DOI:** 10.1186/s12936-025-05774-z

**Published:** 2026-01-06

**Authors:** Ronald Carshon-Marsh, Richard Wen, Thomas Kai Sze Ng, Rajeev Kamadod, Isaac Bogoch, Susan J. Bondy, Theodore J. Witek, Prabhat Jha

**Affiliations:** 1 Dalla Lana School of Public Health, University of Toronto, Toronto, ON M5T 3M7 Canada; 2 Centre for Global Health Research, Unity Health Toronto, Toronto, ON M5B 1W8 Canada; 3 Divisions of General Internal Medicine and Infectious Diseases, Department of Medicine, Toronto General Hospital, University Health Network, University of Toronto, Toronto, ON M5S 3H2 Canada

**Keywords:** Verbal autopsy, Minimally invasive tissue sampling, Malaria, Cause of death, Large language model, GPT-4o, InSilicoVA, InterVA-5, Sub‐Saharan Africa

## Abstract

**Background:**

Malaria, a preventable parasitic disease, causes most child deaths in sub-Saharan Africa (SSA). Reliable cause-of-death data are essential to evaluate progress toward the national and global malaria control goals. However, civil registration and vital statistics are often weak and incomplete in many low- and middle-income countries. In such circumstances, verbal autopsy (VA) provides an alternative means of mortality surveillance. In some settings, VA has been paired with Minimally Invasive Tissue Sampling (MITS) to obtain detailed biological confirmation of the causes of death. Here, we compare malaria-attributed and all-cause mortality among children younger than five years in six SSA countries, using three computer models (GPT-4o, InSilicoVA, and InterVA-5) to assign causes of death, against MITS as the reference standard.

**Method:**

We examined 3129 under-five deaths enrolled in six Child Health and Mortality Prevention Surveillance (CHAMPS) country sites in SSA between December 2016 and December 2022. Contrived free-text narrative summaries were generated for each record and coded into International Classification of Diseases (ICD-10) codes by GPT-4o. InSilicoVA and InterVA-5 outputs, provided in the World Health Organization 2016 VA codes, were harmonized to ICD-10 for comparison. The primary comparison was the underlying cause of death in VA models and MITS.

**Results:**

Sierra Leone had the highest proportion of post-neonatal deaths attributed to malaria at 30.3% (67/221), followed by Kenya at 17.3% (42/243), then Mozambique at 13% (18/138) and Mali at 5.5% (3/55) as defined by MITS. No malaria-attributable deaths were observed in neonates and stillbirths. GPT-4o correctly classified 60 (46.2%) of 130 malaria deaths, compared with 39 (30.0%) for InSilicoVA and 30 (23.1%) for InterVA-5. At the population level, the GPT-4o model achieved a higher cause-specific mortality fraction accuracy (0.36) compared to InSilicoVA (0.07) and InterVA-5 (0.08). GPT-4o performed comparatively better in attributing malaria, HIV/AIDS, and diarrhoeal diseases compared to other communicable diseases.

**Conclusion:**

GPT-4o demonstrated superior performance over probabilistic VA models in identifying malaria-attributed deaths. National vital registration authorities and health ministries should consider integrating large language model-driven tools into their VA systems to enhance diagnostic precision. While less practicable at scale, focal and periodic MITS comparisons are useful for improving verbal autopsy systems. National mortality data are essential to track progress in reducing childhood deaths from malaria and other conditions.

**Supplementary Information:**

The online version contains supplementary material available at 10.1186/s12936-025-05774-z.

## Introduction

Malaria is a preventable and treatable parasitic disease that continues to cause substantial childhood mortality across sub-Saharan Africa (SSA). The World Health Organisation (WHO) estimated the global number of malaria deaths in 2023 as 0.6 million, almost all of which were in African children [1]. The WHO Global Technical Strategy (GTS) for malaria 2016–2030 calls for endemic countries to reduce malaria mortality rates and case incidence by at least 75% by 2025 and at least 90% by 2030 compared to their 2015 baseline [2]. To evaluate progress in reducing malaria incidence and mortality towards GTS targets, accurate and comprehensive malaria mortality data are crucial at the global, national and subnational levels [3, 4].

However, civil registration and vital statistics are often weak and incomplete in many low- and middle-income countries (LMICs), particularly in Africa [4, 5], where the malaria burden is high. A substantial proportion of deaths in Africa do not occur in hospitals but rather at home, where medical attention and information on causes of death are lacking, resulting in misclassification or underreporting of malaria-specific mortality. This challenge continues to hamper the accurate measurement of malaria-specific mortality rates at the population level. Due to this lack of precise information on the cause of death (CoD), LMICs continue to rely on Verbal Autopsy (VA). Minimally Invasive Tissue Sampling (MITS) is a feasible alternative to Complete Diagnostic Autopsy (CDA) to provide detailed biological information on the causes of death. However, MITS is resource-intensive, infrastructure-dependent, and cannot be scaled nationally, in contrast to VA [4, 6].

VA methodology is universally accepted as a practical and scalable method for CoD ascertainment in resource-constrained settings. It has high cultural acceptability, is cost-effective and generates population-level cause-of-death data [3, 6]. Many LMICs, including Sierra Leone and Mozambique, have adopted nationally representative mortality surveillance platforms to generate cause-specific mortality fractions (CSMFs). However, VA has its inherent limitations, such as subjective interpretation by interviewers and physicians, reliance on incomplete or biased information from relatives and caregivers, limited diagnostic accuracy, and inability to differentiate *Plasmodium falciparum* infection from other pathogens with overlapping syndromes [3, 4].

The Child Health and Mortality Prevention Surveillance (CHAMPS) network has been conducting standardized mortality surveillance using MITS in seven countries, predominantly in Africa, to address critical gaps in CoD attribution since 2015 [7]. In the MITS procedure, post-mortem samples of key organs acquired through needle biopsy are thoroughly investigated using histopathological and microbiological techniques, offering a feasible alternative to CDA [7–9]. A recent systematic review of validation studies [4] demonstrated that MITS compared with CDA diagnoses had an overall concordance rate ranging from 68 to 90%, with the highest concordance seen in deaths due to infectious diseases and malignant tumors. When compared to VA, MITS demonstrated better accuracy in diagnosing malaria‑attributed deaths, particularly in hospital settings [4, 9, 10]. However, MITS is not scalable for population-wide surveillance, and its applicability is further limited in capturing malaria deaths occurring outside health facilities [4]. In fact, rural or unattended deaths at home differ substantially in age, educational level, pathogen distribution and etiology from those that occur in urban hospitals [11–14]. A case study and an article from CHAMPS indicate that only about 12 to 13% of all MITS-performed deaths occurred in the community [15, 16]. Most deaths that undergo MITS are hospital deaths; thus, it is difficult to have a reliable “gold” standard to validate VA [11, 13]. Given substantial operational constraints that limit its scalability, MITS is more appropriately suited as an evaluation and comparison tool than as a population-wide surveillance method.

Few published studies have directly compared VA findings against MITS. Empirical evidence is particularly scarce for validation studies comparing VA-derived paediatric malaria mortality data against MITS-confirmed diagnoses. Recent advances in artificial intelligence (AI) have shown considerable promise for improving cause-of-death attribution; however, empirical evidence remains scarce for mortality surveillance applications integrating AI-based VA models, including large language models such as the Generative Pre-trained Transformer-4o model (GPT-4o).

We conducted an observational study using collected CHAMPS data to evaluate the performance of three verbal autopsy models (GPT-4o, InSilicoVA and InterVA-5), comparing them to MITS, a reference standard. This design provides an opportunity to assess both the diagnostic accuracy of emerging artificial intelligence–based approaches and the reliability of established probabilistic models.

## Methods

### General setting

The CHAMPS network conducts standardized mortality surveillance for stillbirths and under-five-year-old children across seven countries in sub-Saharan Africa and South Asia (https://champshealth.org/) [7, 9]. This study included six African countries: Ethiopia, Kenya, Mali, Mozambique, Sierra Leone, and South Africa. Although these sites are not fully representative of all sub-Saharan Africa, they span distinct geographic regions and capture variation in gross domestic product, human development index, life expectancy, and malaria burden.

### Specific settings and study sites

Across the six countries included in this analysis, CHAMPS operates many geographically defined study sites. In Mali, the sites are in Bamako (Djicoroni Para and Banconi). In Ethiopia, surveillance was conducted in Kersa, Harar and Haramaya. In Sierra Leone, the sites are in Makeni (Bombali Shebora and Bombali Siari Chiefdoms) and Bo (Kakua and Tikonko Chiefdoms). Manhiça and Quelimane are the surveillance sites in Mozambique. In Kenya, the study sites are Siaya County (Karemo division) and Kisumu County (Manyatta division), while in South Africa, data were collected from Soweto and Thembelihle sites [7, 9, 17]. CHAMPS site initiation occurred in a phased manner, beginning with Mozambique in 2016; South Africa, Kenya, and Mali in 2017; and Sierra Leone and Ethiopia in 2019 [9].

### Study population and period

Children younger than five years who died at CHAMPS sites in the six sub-Saharan African countries described above and who underwent both VA and MITS with parental or guardian consent were enrolled and studied. Deaths were categorized as stillborn babies, neonates (< 28 days old), infants (28 days to < 12 months old), and children (12 months to < 60 months old). The study period covered deaths recorded between December 2016 and December 2022.

### MITS procedure, analysis, and cause of death determination

The MITS procedure involves disinfection of the deceased's body surface, followed by the post-mortem collection of blood, cerebrospinal fluid (CSF), stool, and nasopharyngeal (NP) swabs and taking necropsy samples using fine needles from solid organs such as the liver, lungs, central nervous system (CNS), heart, spleen, and kidneys. These samples are analysed through microbiological and histopathological techniques as described previously [7, 9]. Clinical data from the hospital, including information on signs, symptoms, and antemortem laboratory tests and imaging from both the child and the mother, if available, were collected. Post-mortem pictures of the child and anthropometric measurements were also collected [7, 9]. The VA tool (WHO 2012 and later 2016 [18]) was used to collect additional information on signs, symptoms, circumstances and narrative of the events leading to death [9].

All enrolled deaths were systematically screened for *Plasmodium falciparum* malaria and other *Plasmodium* species using Rapid Diagnostic Tests (RDTs), thick and thin blood smear microscopy, and real-time quantitative polymerase chain reaction (qPCR) assay.

All the available data, including clinical, laboratory (microbiological and histopathological), sociodemographic, and VA narratives, were compiled into standardized Determination of Cause of Death (DeCoDe) packages and reviewed by a panel of experts [9]. These panels comprised paediatricians, obstetricians, public health specialists, pathologists, and microbiologists who assigned the underlying cause of death as appropriate. The intermediate, immediate and even the contributory CoD were assigned if applicable.

### Verbal autopsy cause of death assignment by computer models

In all study sites in the six countries, VA data were collected from a family member or someone closely associated with the deceased by trained interviewers using the WHO 2016 VA instrument [7, 18]. In general, causes of death from VA have been assigned either by physician review or by computer-based models.

The InterVA-5 model used in this study was substantially built upon the InterVA-4 model and was published in 2019 [19, 20]. The Inter VA-4 model utilizes clinical expert-defined conditional probabilities of observing each symptom given a particular CoD and applies Bayes’ rule to determine the most likely CoD [21].

InSilicoVA, also included in this study, employs a Bayesian framework that utilizes both reported and unreported symptoms to estimate cause-specific probabilities. The model is estimated using Markov chain Monte Carlo simulations and, like InterVA-5, has been widely implemented in mortality surveillance [22]. However, both InterVA-5 and InSilicoVA are constrained by reliance on structured symptom questionnaires and do not incorporate open narrative text, which may contain valuable contextual information such as symptom chronology or care-seeking behaviours [23].

Large language models (LLMs) offer potential improvements in this regard. ChatGPT, developed by OpenAI, introduced the capacity to process and interpret free-text inputs with increasing accuracy across successive generations. We used one of the latest multimodal LLMs from OpenAI called GPT-4omni (or GPT-4o), released on May 13, 2024 [24]. When GPT-4o was compared with ten top LLMs using metrics such as throughput, response time, and latency, GPT-4o demonstrated clear superiority [25].

### VA and MITS records from CHAMPS

The CHAMPS network provided 4485 mortality records that had undergone MITS and were reviewed through the DeCoDe panel, with International Classification of Diseases, 10th Revision (ICD-10) codes assigned. Country-specific contributions were as follows: Bangladesh (549 records), Sierra Leone (653 records), South Africa (1,030 records), Mozambique (995 records), Ethiopia (361 records), Kenya (658 records), and Mali (239 records).

Of these, 3,566 deaths had complete VA questionnaires (excluding the narrative). The WHO 2016 VA questionnaire was used in 3,494 records, and the WHO 2012 VA questionnaire in the remaining 72 records. These 72 records (35 stillbirth and neonatal records and 37 infant and child records from Mozambique) were excluded because the old WHO 2012 VA questionnaire forms, which had a relatively different structure, were used instead of the 2016 WHO VA instrument.

**Fig. 1:**
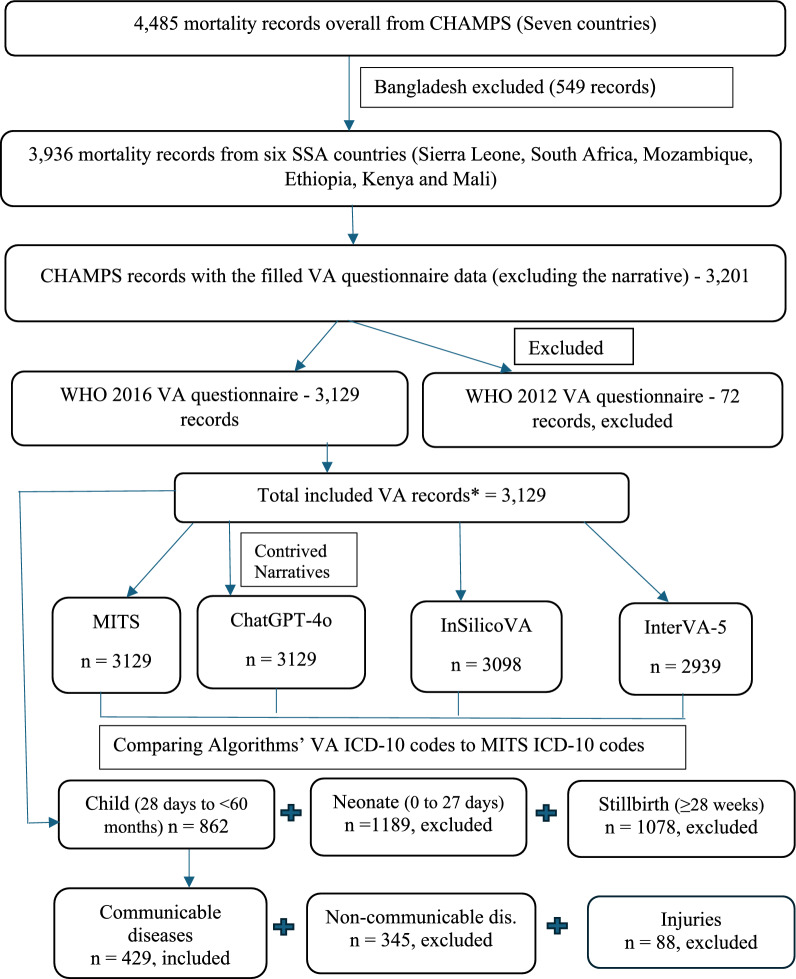
Study design and inclusions/exclusions. *Total included VA records = 3,129: 534—Sierra Leone, 735—South Africa, 705—Mozambique, 345—Ethiopia, 581—Kenya & 229—Mali. VA: Verbal Autopsy; MITS: Minimally Invasive Tissue Sampling; WHO: World Health Organization

Since this research focused on SSA, records from Bangladesh were excluded. The final analytic dataset comprised 3,129 records (534 from Sierra Leone, 735 from South Africa, 705 from Mozambique, 345 from Ethiopia, 581 from Kenya and 229 from Mali). The flow of records through inclusion and exclusion criteria is shown in Fig. 1.

### Input data and preprocessing for GPT-4o model

The de-identified Excel data received from CHAMPS were without the VA narratives. The GPT-4o model requires free-text narrative summaries to determine the underlying cause of death. To facilitate analysis, synthetic or contrived narratives were generated using structured variables from the 2016 WHO VA instrument. Two types of text prompts (user and system prompts) were generated as input to instruct the model to assign COD based on the open narratives. A narrative template was then developed to systematically extract information from the CSV files and transform it into free text. See annex 1 for more details.

The following narrative template was used to generate the VA narrative:

"Narrative template": "{system_prompt}\n\n{user_prompt}\n\nThis {sex} child from {country} is {age} old or was born on {dob} and died on {dod}. The child was sick for {sick_days} days and died at the {facility}. The child had the following signs and symptoms: {list_of_symptoms}. It was a {case_type_desc} death.\n\n"

Where dob = date of birth, dod = date of death and case_type_desc = Age group of the CHAMPS Case.

In addition to the standard template, more information on whether the baby cried, breathed and suckled immediately at birth or not was added for early neonatal deaths. Also, the results of cases that tested positive for Tuberculosis, Human Immunodeficiency Virus (HIV)/Acquired Immune Deficiency Syndrome (AIDS) and malaria were included in the narrative. The ID codes from the 2016 WHO VA questionnaire were matched with their signs and symptoms.

A total of 3,129 VA narratives were generated. Each narrative was manually reviewed to ensure missing data, such as 'NA', '99', etc., were removed and corrected, as well as any age/category mismatch.

### InSilico VA and InterVA-5 records and preprocessing

CHAMPS also provided 4,005 records coded by both InSilicoVA and InterVA-5 in the WHO VA 2016 codes. Each record contained up to three causes of death (prioritized as cause 1, cause 2, and cause 3) with associated probabilities. Following the exclusion of Bangladesh records, 3,474 records remained for InterVA-5 and InSilicoVA. All the WHO VA 2016 codes in all three sets of causes were converted to ICD-10 codes using a mapping file (https://openmortality.org/data/wva2016_icd10_v1).

### Output data

The 3,129 open free-text contrived narratives were processed and coded by OpenAI GPT-4o, thus yielding the 3,129 ICD-10 codes. After the InterVA-5 and InSilicoVA WHO VA 2016 codes were converted into ICD-10 output codes, the data were superimposed with the GPT-4o data, matching them by their CHAMPS ID numbers or record identifiers. Of the 3,129 GPT-4o-coded records, 3,098 (99%) aligned with InSilicoVA and 2,939 (94%) with InterVA-5. The 3,129 MITS ICD-10 CoD were comprised of 862 children (1 to < 60 months), 1189 neonates (< 28 days) and 1078 stillbirths (≥ 28 weeks).

### Comparisons and statistical analysis

The diagnostic performances of the three models, GPT-4o, InterVA-5, and InSilicoVA, were evaluated with metrics at both the individual and population levels by comparing their ICD-10 CoD codes to MITS as the reference standard. The accuracy of the VA model at the individual level was evaluated using the Partial Chance Corrected Concordance (PCCC) by comparing the CoD established by MITS with the most probable CoD provided by the VA model, adjusting for random chance. Values of PCCC close to 1 indicate strong alignment with MITS, whereas values near 0 indicate poor agreement. See annex 2 for more details.

The Cause Specific Mortality Fraction (CSMF) accuracy was used to evaluate the VA models at the population level in comparison to MITS CoD, adjusting for maximum total error from the worst model. It is scaled from zero to one and can generalize a method's CSMF estimation capability regardless of the number of causes [26]. A value of one means no error in the predicted CSMFs or the GPT-4o model VA CoD distribution completely matched the MITS CoD distribution, and a value of zero means the method is equivalent to the least accurate method of assigning cause fractions, or it did not match the distribution at all [26]. Both PCCC and CSMF accuracy have been well-studied; when they were assessed across many test datasets with widely varying CSMF composition, they objectively evaluated the performance of VA models in assigning cause of death [26].

The diagnostic performance of the VA model to identify malaria as the underlying CoD against MITS was evaluated in terms of Cohen’s Kappa, sensitivity, specificity, positive predictive value (PPV) and negative predictive value (NPV). The sensitivity of the VA model for malaria is the proportion of deaths with a CoD correctly identified as malaria out of all those who truly died from malaria, according to MITS. The specificity of the VA model is the proportion of deaths with a CoD correctly identified as not malaria among those who truly did not die from malaria according to MITS. The PPV of the VA model in relation to MITS measures the proportion of positive VA diagnoses, while the NPV of the VA model measures the proportion of negative VA diagnoses that are confirmed by MITS [27]. Cohen’s kappa is a robust statistic useful for interrater reliability testing [28]. See annex 2 for more details on these metrics. To visualize VA model-MITS differences in individual causes of death classification for major communicable diseases, alluvial diagrams were generated.

The metrics were calculated for children (1 to < 60 months). Neonatal deaths were excluded from the analysis since no malaria death was identified as the underlying CoD in the 1,189 neonatal deaths. For consistency across methods, only the underlying CoD was considered for verbal autopsy models, while the underlying CoD determined by MITS was used as the reference.

Data analysis was performed using the statistical software R v4.4.2 (R Core Team, Vienna, Austria). The packages library glue and yam were used to generate the prompts and the VA narrative, ggalluvial v0.12.5 for alluvial diagrams, and tidyverse v2.0.0 for data processing and plotting.

### Ethical review and approvals

Ethics approval (RIS Human Protocol Number: 00047427) was obtained from the Health Sciences Research Ethics Board (REB) of the University of Toronto on the 14th of November 2024. There was also REB approval from the Centre for Global Health Research, St. Michael’s Hospital, Toronto (Unity Health Toronto REB, Statistical Alliance for Vital Events (SAVE) under REB #15–231) covering this study. The CHAMPS network obtained institutional and national ethical approvals in all participating countries before data collection.

## Results

A total of 3,129 VA records with contrived free-text narratives were coded using the OpenAI GPT-4o, thus generating corresponding ICD-10 codes. Of these, there were 534 VA records from Sierra Leone, 735 from South Africa, 705 from Mozambique, 345 from Ethiopia, 581 from Kenya and 229 from Mali. The dataset included 862 child records, 1,189 neonates, and 1,078 stillbirths. No malaria-attributable deaths were identified among stillbirths and neonates. Among children aged 1–59 months, 429 deaths (49.8%) were attributed to communicable diseases, 345 (40.0%) to non-communicable diseases, and 88 (10.2%) to injuries (Fig. 1).

### Malaria-specific mortality

Based on CHAMPS MITS' underlying CoD, 130 deaths were attributed to malaria, representing the leading communicable disease and accounting for 30% (130 of 429) of all infectious disease deaths. Of these, 31 (24%) occurred in infants aged 1–11 months, and the remainder occurred in children (12–59 months). Sierra Leone accounted for 67 malaria deaths (52%), followed by Kenya with 42 malaria deaths (32%). The proportion of post-neonatal deaths attributed to malaria by country is shown in Table 1.

**Table 1:** Proportion of CHAMPS deaths attributed to malaria by country defined by MITS

Country	Total MITS records	Number of malaria/overall deaths ages 1–59 months	Percentage of malarial deaths at 1–59 months (%)
Sierra Leone	534	67/221	**30.3**
Kenya	581	42/243	**17.3**
Mozambique	705	18/138	**13.0**
Mali	229	3/55	**5.5**
Ethiopia	345	0/25	**0.0**
South Africa	735	0/180	**0.0**
Total	3129	130/862	15.1

CHAMPS: Child Health and Mortality Prevention Surveillance; MITS: Minimally Invasive Tissue Sampling. Bold values indicate percentage of post-neonatal (1-59 months) malarial deaths by country

Based on the MITS underlying CoD, malaria accounted for 30.3% (67/221) of the post-neonatal deaths from Sierra Leone, 17.3% (42/243) in Kenya, 13% (18/138) in Mozambique and 5.5% (3/55) in Mali. No malaria-attributed death was identified in Ethiopia and South Africa. When applying the VA models against MITS-confirmed malaria deaths, GPT-4o correctly classified 60 (46.2%) of 130 malaria deaths, compared with 39 (30.0%) for InSilicoVA and 30 (23.1%) for InterVA-5.

### Malaria-specific mortality metrics

All three VA models demonstrated a fair agreement with MITS in attributing malaria as an underlying CoD. Cohen’s Kappa values were 0.38 for GPT-4o, 0.31 for InSilicoVA, and 0.30 for InterVA-5 (Table 2). The sensitivity levels for the VA models were low compared to MITS. GPT-4o achieved higher sensitivity than InSilicoVA and InterVA-5, correctly identifying 46% (60/130) of malaria deaths confirmed by MITS, but missed more than half (54%). InSilicoVA and InterVA-5 identified 30% and 23% of malaria deaths, respectively. The specificities of GPT-4o, InSilicoVA, and InterVA-5 were consistently high across models at 0.97, 0.98, and 0.99, respectively. The GPT-4o model correctly identified 97% of the deaths that were not due to malaria according to MITS. All three models are very good at ruling out malaria when it is not the true cause.

**Table 2:**
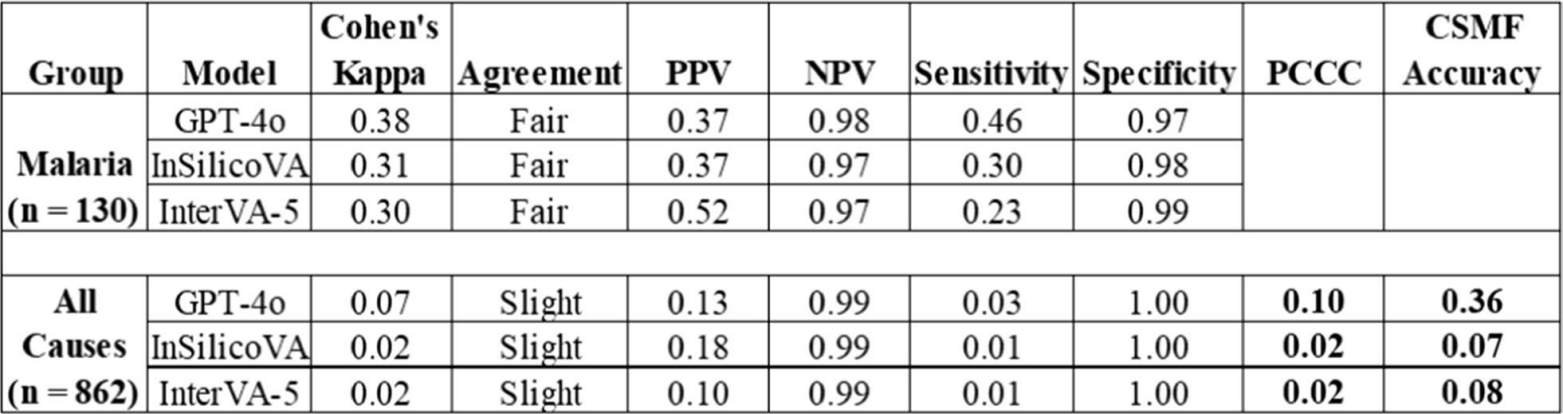
Malaria and child–specific mortality metrics

PPV: Positive Predictive Value; NPV: Negative Predictive Value; PCCC: Partial Chance Corrected Concordance; CSMF Accuracy: Cause Specific Mortality Fraction Accuracy

Positive Predictive Values were low, ranging from 0.37 with GPT-4o to 0.52 with InterVA-5. This means that among the deaths, the GPT-4o model labelled malaria, only 37% were truly malaria according to MITS. The model's “malaria death” predictions have low reliability; only 1 in 3 predictions is correct. However, for InterVA-5, about 1 in 2 predictions is correct. Negative Predictive Values exceeded 0.96 for all models, suggesting strong reliability in excluding malaria as a cause of death (Table 2).

### Cause of death assignment of the GPT-4o model at the individual level among children who died from malaria

**Fig. 2:**
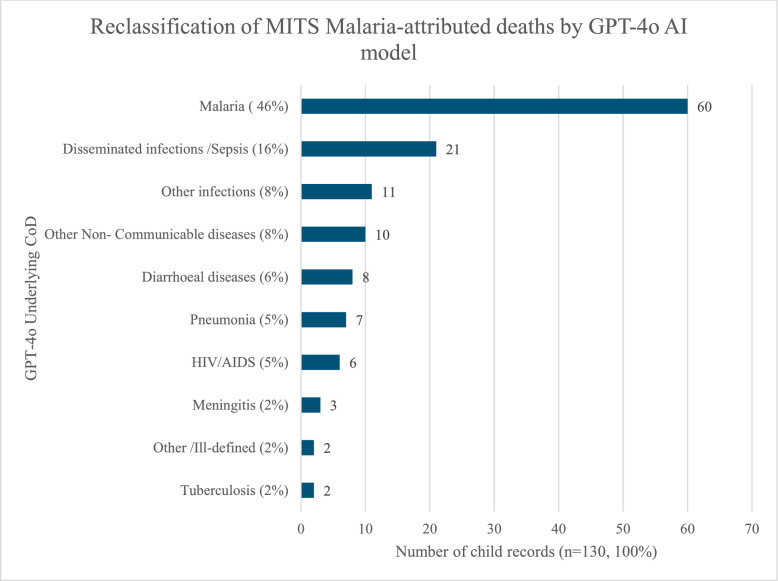
Reclassification of Malaria-attributed deaths by the GPT-4o AI model in comparison to MITS. Figure shows the Individual-level GPT-4o model performance for child (post-neonatal) malaria-attributed cause of death with reference to MITS. The bars show the various CoD attributed by the model

Of the 130 deaths attributed to malaria by MITS, 46% (60/130) were correctly classified as malaria by GPT-4o. The GPT-4o model reclassified 16% of the malaria-attributed deaths by MITS as disseminated infections or sepsis (see Fig. 2). Reclassification also occurred as ‘other infections’ (8%), other non-communicable diseases (8%), followed by diarrhoeal diseases (6%), then pneumonia (5%) and HIV (5%). Overall, the GPT-4o model outperformed InSilicoVA (30%) and InterVA-5 (23%) in correctly classifying malaria-attributed deaths.

### Overall performance at the individual and population level

At the individual level in the child age group (1–59 months), the PCCC shows only slight agreement between MITS and the GPT-4o (0.10), InSilicoVA (0.02), and InterVA-5 (0.02) models, as shown in Table 2. Even though the specificities and NPVs were very high, the sensitivities and the PPVs were very low.

At the population level, the GPT-4o model achieved a CSMF Accuracy of 0.36, which indicated that 36% of the cause-specific distribution was correctly captured compared to MITS. This is a modest performance, suggesting considerable reclassification between GPT-4o VA CoD and MITS CoD. The InSilicoVA and InterVA-5 models exhibited an abysmal performance, with accuracies of only 7% and 8%, respectively, indicating that their predictions were almost entirely inaccurate in terms of cause distribution. However, GPT-4o clearly had the best performance of all three VA models at the population level in the child age group.

### Cause of death assignment of the GPT-4o, InSilicoVA and InterVA-5 models for communicable diseases compared to MITS

There was a total of 429 communicable disease records based on the MITS underlying CoD, with the most prevalent being malaria, accounting for 30% of the mortality records, followed by pneumonia (20%), HIV/AIDS (19%), diarrhoeal diseases (14%), disseminated infections (9%), and ‘other infections’ (5%).

### VA cause of death assignment using GPT-4o

**Fig. 3:**
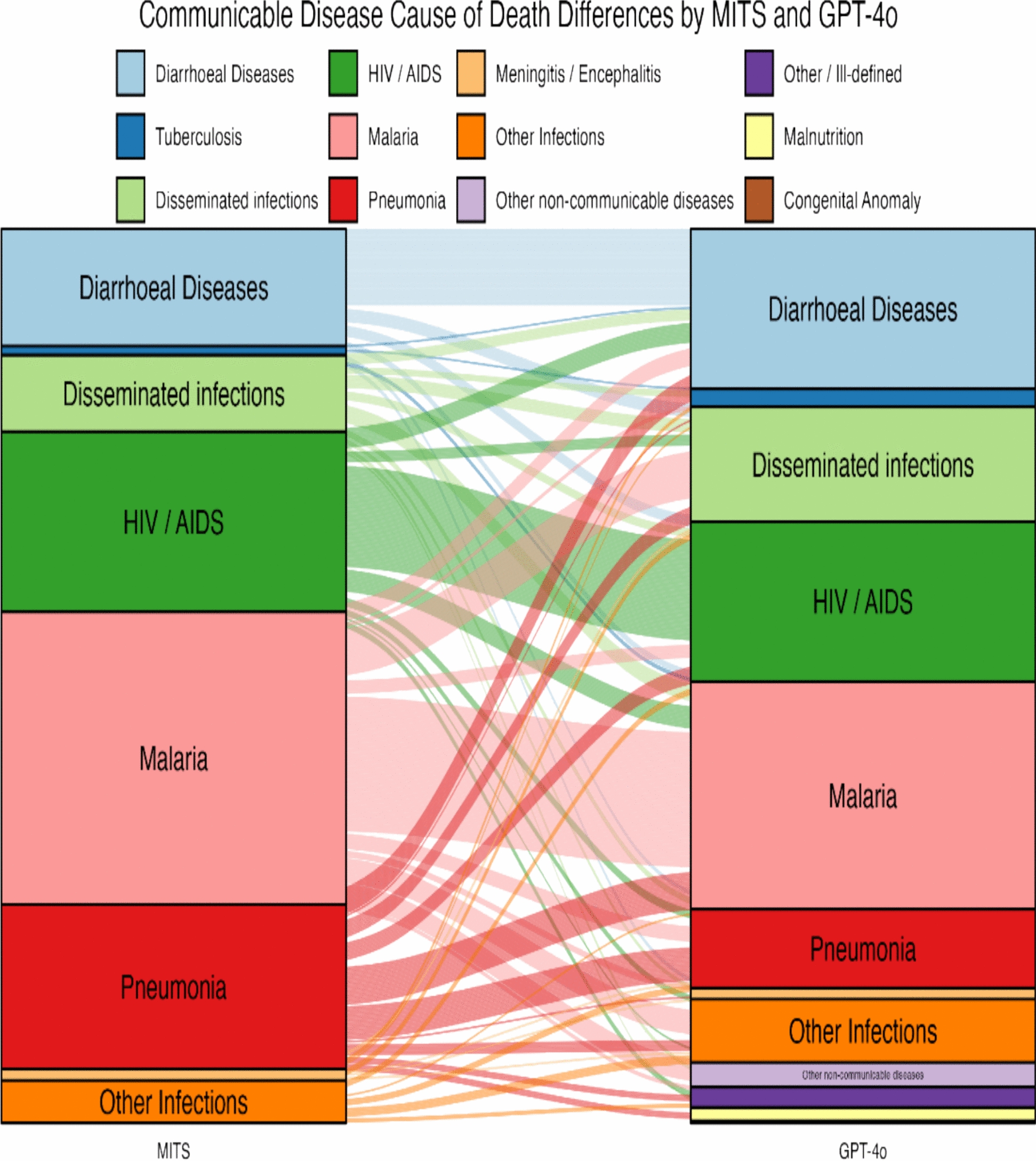
Alluvial diagram showing differences in individual causes of death as established by MITS and the GPT-4o AI model among children aged 1–59 months who died from communicable diseases. The stacked blocks represent causes of death (CoDs) determined by MITS (left) and by the GPT-4o model (right), with their size proportional to cause-specific mortality fractions (CSMFs). The branches between blocks illustrate differences in the composition of CoDs between the two methods, with their thickness proportional to the number of cases contained in both blocks connected by the branch. Each CoD is represented by a different colour, consistent across both diagnostic methods. The colour of the branches indicates the actual cause of death as determined by MITS. Concordant cases between MITS and the GPT-4o AI model are shown as branches connecting blocks of the same colour, while reclassified cases are represented by branches linking blocks of different colours

GPT-4o showed variable performance across these conditions. As mentioned earlier, 46% of malaria-attributed deaths were correctly classified as malaria by GPT-4o in comparison to MITS. The GPT-4o model—MITS differences in individual causes of death classification for communicable diseases have been shown in the alluvial diagram (Fig. 3). Only 17% of the pneumonia-attributed deaths by MITS were correctly classified by GPT-4o, while 20% of the pneumonia-attributed deaths were reclassified as malaria, and 14% as diarrhoeal diseases (Annex 3). HIV/AIDS deaths were correctly attributed in 56% of cases, although 12% were reclassified as malaria and 11% as diarrhoeal disease deaths. For diarrhoeal disease, GPT-4o achieved 57% concordance and reclassified diarrhoeal disease deaths as disseminated infections (10%) and HIV (8%). Disseminated infection deaths were correctly classified in 15% of cases but were often reclassified as malaria (21%) or diarrhoeal disease (13%). GPT-4o thus performed comparatively better in attributing malaria, HIV/AIDS, and diarrhoeal diseases, with weaker performance for pneumonia and disseminated infections.

### VA cause of death assignment using InSilicoVA

InSilicoVA correctly classified 30% of malaria deaths, but reclassified 20% as other infections, 19% as pneumonia, 14% as other/ill-defined, and 9% as meningitis, compared to MITS. For pneumonia, 56% of deaths were correctly attributed, with 13% reclassified as diarrhoeal diseases and 10% as malaria. Only 26% of HIV/AIDS deaths were correctly identified, with frequent reclassification as diarrhoeal disease (21%) or pneumonia (20%). In comparison to MITS, 53% of diarrhoeal disease-attributed deaths were correctly classified as diarrhoeal disease; however, 17% and 13% were reclassified as pneumonia and ‘other infections’, respectively (see Annex 4 for the alluvial diagram and Annex 5 for the reclassification tables). Deaths due to disseminated infections by MITS were reclassified mostly as pneumonia (26%), malaria (15%) and ‘other infections’ (15%) by InSilicoVA. InSilicoVA demonstrated relatively better performance for pneumonia and diarrhoeal disease attribution compared with other infectious diseases.

### VA cause of death assignment using InterVA-5

Compared to MITS, 23% of the malaria-attributed deaths were correctly classified by InterVA-5. Deaths were frequently reclassified by InterVA-5 as other/ill-defined (19%), meningitis (14%), pneumonia (14%) and ‘other infections’ (12%). Pneumonia was correctly classified in 23% of the deaths compared to MITS by InterVA-5. Deaths were incorrectly classified as diarrhoeal diseases (20%), other infections (14%), and meningitis (9%). For HIV/AIDS, 25% of deaths were correctly classified, while 25% were reassigned to diarrhoeal disease and 12% to other infections. InterVA-5 achieved 52% accuracy in classifying diarrhoeal disease, with substantial reclassification into other/ill-defined (17%) and other infections (10%). Disseminated infection deaths were largely reassigned to diarrhoeal diseases (23%), other infections (18%), and pneumonia (13%) (see Annex 6 for the alluvial diagram and Annex 7 for the reclassification tables). Overall, InterVA-5 performed comparatively better for diarrhoeal disease attribution but poorly for malaria and pneumonia.

## Discussion

In this multi-country comparison study across six sub-Saharan African sites in the CHAMPS network, the performance of three VA models (GPT-4o, InterVA-5, and InSilicoVA) was assessed against MITS as the reference standard for attributing malaria and all-cause mortality among children under five years of age. The present study demonstrated that malaria accounted for nearly one in five post-neonatal child deaths in malaria-endemic sites, irrespective of the variability in malaria epidemiology and transmission settings, underscoring its continued importance as a leading cause of under-five mortality in the region. While GPT-4o outperformed the two established VA models (InSilicoVA and InterVA-5) in correctly attributing malaria deaths, its sensitivity and positive predictive value remain limited, highlighting challenges in relying on VA alone for cause-specific mortality surveillance in high-burden settings.

The geographic heterogeneity in malaria-attributed mortality observed in this study mirrors epidemiological trends across the continent. Malaria accounted for substantial post-neonatal deaths in Sierra Leone, Kenya and Mozambique, but was virtually absent in Ethiopia and South Africa. The absence of malaria mortality in South Africa aligns with its low national prevalence estimate [29]. These findings corroborate earlier reports of Ogbuanu et al. [9], who also reported higher malaria mortality along the causal chain in Sierra Leone and Kenya. Our malaria mortality figures were lower since only the underlying CoD was used to compare VA and MITS. This gradient reinforces the value of MITS in comparing VA across diverse transmission settings. As demonstrated in our earlier systematic review [4], these differences are shaped by variability in malaria epidemiology, environmental conditions, demographic structure, seasonal variations, health system differences, bias and diagnostic criteria.

The relatively high specificity (> 96% across all models) indicates that VA-based models can reliably exclude malaria as a cause of death. However, their limited sensitivity constrains their utility for quantifying malaria mortality at the population level, raising the risk of systematic underestimation. This could result in systematic biases in mortality surveillance and programmatic decision-making. The results of VA studies with high specificity in malaria endemic areas are largely plausible [4, 30].

GPT-4o correctly identified about 50 to 100% more malaria deaths compared to InSilicoVA and InterVA-5, even without biological data or testing, suggesting that LLMs may offer a step-change improvement in processing VA narratives. However, this level of performance remains insufficient for use in isolation. In the Healthy Sierra Leone project (HEAL-SL), physician coders are supported with LLMs, notably ChatGPT-4 and probabilistic models for CoD determination. Nevertheless, over half of malaria deaths were incorrectly classified, most commonly as disseminated infections, diarrhoeal diseases, HIV/AIDS, or pneumonia, reflecting overlapping clinical syndromes in childhood infectious diseases and the inherent limitations of symptom-based mortality attribution. However, GPT-4o showed comparatively higher concordance in attributing malaria, HIV/AIDS, and diarrhoeal diseases, while InSilicoVA demonstrated relatively better performance for pneumonia and diarrhoeal disease attribution, and InterVA-5 also showed modestly higher concordance for diarrhoeal disease attribution. Despite these limitations, GPT-4o achieved substantially higher CSMF accuracy than InSilicoVA and InterVA-5 (0.36 vs 0.07 and 0.08, respectively), supporting its potential to improve population-level mortality estimation especially malaria surveillance. These findings are noteworthy given that accurate population-level estimates of cause-specific mortality are central to public health planning. Unlike probabilistic models, GPT-4o was able to incorporate contrived narrative text, potentially capturing subtle information missed by symptom-only approaches. Nevertheless, its performance remains limited by the absence of full free-text VA narratives in the CHAMPS dataset and by the clinical overlap between malaria and other common infections in children.

Our study extends recent work by Wen et al. [31], who found that GPT-based models achieved higher concordance with physician-coded VA data compared to probabilistic models in Sierra Leone. Together, these studies suggest that generative AI models can capture subtle diagnostic signals missed by symptom-only models. However, our study grounds this advancement in a direct comparison with MITS, providing a more robust benchmark of its true diagnostic accuracy.

Our findings are consistent with earlier comparison studies, which have shown that VA-based models have lower concordance at the individual level when compared with MITS and CDA methods, but relatively higher at the population level [4, 9, 10, 32, 33]. While MITS offers high diagnostic precision, operational and cultural constraints limit its scalability across routine mortality surveillance. In contrast, VA remains the only feasible method for large-scale cause of death surveillance in resource-limited settings or countries with weak CRVS systems. Thus, improving the accuracy of VA interpretation is critical. The introduction of generative AI models capable of processing unstructured narrative text appears to improve discriminatory ability, particularly in distinguishing malaria from other communicable diseases, but further refinements are required before such models can be scaled for routine surveillance. It is important to note that the quality of the VA narrative strongly influences the AI model's performance; detailed, context-rich family accounts substantially improve the AI model’s discriminatory performance, whereas brief or poorly documented narratives diminish its diagnostic reliability.

Computer-coded verbal autopsy models, including emerging LLM approaches, reduce reliance on physician reviewers and offer substantial advantages in scalability, standardization, and efficient use of both structured and narrative data. LLMs add further strengths in narrative understanding, linguistic flexibility, contextual reasoning, and adaptability [34, 35]. Despite their potential to interpret complex narratives, limitations of LLM for VA CoD assignment include limited contextual understanding or epidemiological grounding, removal of human interpretive judgment, variable reproducibility, limited transparency, susceptibility to bias, regulatory concerns, and challenges in distinguishing infection from causation [36].

The exclusion of neonates from malaria-specific validation, based on the rarity of malaria in this age group, further reflects the need for age-stratified approaches in VA interpretation. Less than a quarter of the malaria deaths occurred in infants aged 1–11 months. In malaria-endemic settings, malaria infection rates are low among infants younger than 6 months, primarily due to the transient protection provided by maternally derived malaria-specific immunoglobulin G (IgG) antibodies acquired in utero, which wane progressively during the first year of life. [37, 38]. With cumulative exposure to malaria parasites through repeated infectious bites from female Anopheles mosquitoes, older children, adolescents, and adults gradually develop partial naturally acquired immunity (NAI) to malarial disease, mediated by increasingly effective, strain-specific immune responses [4, 9]. Younger children still developing NAI remain highly susceptible to clinical malaria disease, thus resulting in high malaria morbidity and mortality among them. This NAI results in clinical tolerance to malaria infections, so a malaria infection detected by RDT or microscopy in a person may not necessarily indicate that malaria is the cause of the illness or death. This challenge in distinguishing between the presence of a malaria infection and the infection being the true cause of death is a key limitation of VA. Postmortem methods such as MITS are important because they enable the detection of malaria parasites within vital organs (including the brain, liver, and spleen) and the characterization of pathological changes consistent with severe malaria, thereby directly linking infection to the pathophysiological processes leading to death.

Our findings hold implications for health policy and practice. First, the superior performance of GPT-4o compared with existing probabilistic models suggests that artificial intelligence can play a complementary role in improving VA analysis. National vital registration agencies and health ministries should begin integrating AI-driven tools into their VA systems to enhance data quality and timeliness. This can be achieved as part of a hybrid surveillance model that also combines innovative AI-driven VA interpretation with periodic MITS comparisons and strengthened CRVS systems. Second, the findings of this study reinforce the urgent need to strengthen cause-of-death surveillance systems across sub-Saharan Africa. Reliable mortality surveillance, underpinned by accurate VA interpretation, is essential for tracking progress toward Sustainable Development Goal 3.2 and the WHO’s GTS for Malaria 2030 targets. Finally, for global health research and development, our findings highlight an urgent need for targeted investment. Donors and research institutions should prioritize funding for the development and localized fine-tuning of AI models on diverse, region-specific datasets.

This study has several strengths. It is a large multi-country study of malaria and other infectious disease mortality attribution, with over 3,100 VA records in comparison to MITS data as the reference standard. The use of multiple VA models, including one of the most recent large language models, provides comparative insights into current and emerging approaches. Additionally, the study spanned diverse epidemiological settings, enhancing generalisability across sub-Saharan Africa.

Nonetheless, this study has some limitations. First, our reliance on contrived VA narratives, reconstructed from structured questionnaires, may not fully capture the richness of true open-ended family accounts, potentially underestimating GPT-4o’s performance. Second, although MITS is a more robust diagnostic tool than VA, it is not without constraints, including incomplete sampling and operational challenges that limit scalability [4]. Third, neonatal deaths were excluded from malaria-specific validation due to their very low probability of malaria attribution, which may limit comparability with broader under-five mortality studies. Finally, despite the large overall sample size, the number of malaria deaths at some sites was low, restricting the precision of some cross-country comparisons.

## Conclusion

This multi-country comparison of verbal autopsy to biologically confirmed causes of death reaffirm malaria as a leading cause of childhood mortality in endemic regions of sub-Saharan Africa. GPT-4o, a large language model, outperformed traditional probabilistic VA approaches in identifying malaria-attributed deaths; however, its sensitivity and predictive accuracy are still insufficient for use as a standalone tool in mortality surveillance. When compared with MITS, all three VA models exhibited very low individual-level and low population-level concordance, although GPT-4o performed comparatively better at the population level, notwithstanding its limitations.

Our findings compel a shift in mortality surveillance strategy. National vital registration authorities and health ministries should consider the integration of AI-driven tools into their VA systems to enhance diagnostic precision and timeliness, while combining innovative AI-driven VA interpretation with periodic MITS comparisons. Such an integrated approach would generate reliable, actionable data essential for guiding effective malaria control policies, allocating resources efficiently, and ultimately accelerating progress toward the elimination of preventable child deaths.

## Supplementary Information

Supplementary Material 1: Annex 1: Input Data and Preprocessing for GPT-4o model. Annex 2: Comparisons and Statistical Analysis.

Supplementary Material 2: Annex 3: Reclassification tables by communicable disease—MITS vs GPT-4o AI model.

Supplementary Material 3: Annex 4: Alluvial diagram showing Communicable disease CoD differences by MITS and InSilicoVA.

Supplementary Material 4: Annex 5: Reclassification tables by communicable disease—MITS vs InSilicoVA model.

Supplementary Material 5: Annex 6: Alluvial diagram showing Communicable disease CoD differences by MITS and InterVA-5. 

Supplementary Material 6: Annex 7: Reclassification tables by communicable disease—MITS vs InterVA-5 model.

## Data Availability

All data relating to the present study are available in this manuscript and the Additional/Supplementary files.

## Abbreviations

AI: Artificial Intelligence
CDA: Complete Diagnostic Autopsy
CHAMPS: Child Health, and Mortality Prevention Surveillance
CoD: Cause of Death
CNS: Central Nervous System
CSF: Cerebrospinal Fluid
CSMF: Cause Specific Mortality Fraction
DeCoDe: Determination of Cause of Death
GPT-4o: Generative Pretrained Transformer-4omni
GTS: Global Technical Strategy for malaria
HEAL-SL: Healthy Sierra Leone study
LLM: Large Language Model
LMIC: Low- and Middle-Income Countries
MITS: Minimally Invasive Tissue Sampling
NAI: Naturally Acquired Immunity
NPV: Negative Predictive Value
PCCC: Partial Chance Corrected Concordance
PPV: Positive Predictive Value
PCR: Polymerase Chain Reaction
RDT: Rapid Diagnostic Test
SSA: Sub-Saharan Africa
VA: Verbal Autopsy
WHO: World Health Organisation
