# Supplementary material for: Comparison of verbal autopsy using a large language model to biologically confirmed causes of death for malaria and other communicable diseases among children in six sub-Saharan African countries
Source: Malar J. 2026 Jan 6;25:77. doi: 10.1186/s12936-025-05774-z (PMC12870146; doi:10.1186/s12936-025-05774-z)
Supplement: Supplementary file 1 — Supplementary Material 1: Annex 1: Input Data and Preprocessing for GPT-4o model. Annex 2: Comparisons and Statistical Analysis. [file 12936_2025_5774_MOESM1_ESM.docx]

**Annex 1**

Input Data and Preprocessing for GPT-4o model

The de-identified Excel data received from CHAMPS were without the VA narratives. The GPT-4o model requires free-text narrative summaries to determine the underlying cause of death. To enable analysis, synthetic or contrived narratives were generated using structured variables from the WHO 2016 VA instrument. Two types of text prompts (user and system prompts) were generated as input to instruct the model to assign COD based on the open narratives. System prompts contained textual instructions to assign the role of a physician ICD-10 coder with expertise in the required country. The following system prompt was used for each record:

"System Prompt": "You are a physician with expertise in determining underlying causes of death in children in {country} by assigning the most probable ICD-10 code for each death using verbal autopsy narratives. Return only the ICD-10 code without description, e.g. B50. If there are multiple icd_10 codes, show one code per line."

User prompts contained textual instructions to perform coding of VA records based on age, sex, and country, followed by the generated narrative of the deceased. The following template was used to generate user prompts for each record:

"User prompt": "Determine the underlying cause of death and provide the most probable ICD-10 code for a verbal autopsy narrative of {age} old {sex} death in {country}:"

A narrative template was then developed to systematically extract information from the CSV files and transform it into free text. The following narrative template was used to generate the VA narrative:

"Narrative template": "{system_prompt}\n\n{user_prompt}\n\nThis {sex} child from {country} is {age} old or was born on {dob} and died on {dod}. The child was sick for {sick_days} days and died at the {facility}. The child had the

following signs and symptoms: {list_of_symptoms}. It was a {case_type_desc} death.\n\n"

In addition to the standard template, more details on whether the baby cried, breathed and suckled immediately at birth or not were added for early neonatal deaths. Also, the results of cases that tested positive for Tuberculosis, Human Immunodeficiency Virus (HIV)/Acquired Immune Deficiency Syndrome (AIDS) and malaria were included in the narrative. The ID codes from the 2016 WHO VA questionnaire were matched with their signs and symptoms.

A total of 3,129 VA narratives were generated. Each narrative was manually reviewed to ensure missing data, such as 'NA', '99', etc., were removed and corrected, as well as any age/category mismatch.

NB: Dob = Date of birth; dod = Date of death; case_type_desc = Age group of the CHAMPS Case

**Annex 2**

Comparisons and Statistical Analysis

The accuracy of the VA model at the individual level was evaluated using the Partial Chance Corrected Concordance (PCCC) by comparing the CoD established by MITS with the most probable CoD provided by the VA model. PCCC is given by the equation below, where k is the number of top COD assignments from the model to consider, N is the number of causes, and C is the fraction of death records where the MITS COD assignment is one of the top COD assignments from the model [26]. The partial chance-corrected concordance for the top k causes, PCCC(k), becomes:


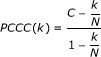


Values of PCCC close to 1 indicate strong alignment with MITS, whereas values near 0 indicate poor agreement.

CSMF accuracy is defined as one minus the sum of all absolute CSMF errors across causes divided by the maximum total error [26]. It is scaled from zero to one and can generalize a method's CSMF estimation capability regardless of the number of causes [26]. A value of one means no error in the predicted CSMFs or the GPT-4o model VA CoD completely matched the MITS CoD, and a value of zero means the method is equivalent to the least accurate method of assigning cause fractions, or it did not match the distribution at all [26].


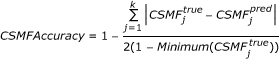


The sensitivity of a VA for a particular cause of death such as malaria is the proportion of deaths with a CoD correctly identified as malaria out of all those who truly died from malaria (according to MITS). In other words, it is the ability of the VA to correctly identify malaria cases that are confirmed by MITS.

Sensitivity = True Positives (TP)​

__________________________________

True Positives (TP)+False Negatives (FN)

The specificity of a VA is the proportion of deaths with a CoD correctly identified as not malaria among those who truly did not die from malaria (according to MITS).

Specificity = True Negatives (TN)​

____________________________________

True Negatives (TN)+False Positives (FP)

The Positive Predictive Value (PPV) of VA in relation to MITS measures the proportion of positive VA diagnoses that are confirmed by MITS.

PPV = True Positives (TP)​

_________________________________

True Positives (TP)+False Positives (FP)

The Negative Predictive Value (NPV) of Verbal Autopsy (VA) in relation to Minimally Invasive Tissue Sampling (MITS) measures the proportion of negative VA diagnoses that are confirmed by MITS.

NPV = True Negatives (TN)​

_________________________________

True Negatives (TN)+False Negatives (FN)

Cohen’s kappa is a robust statistic useful for either interrater or intrarater reliability testing. Similar to correlation coefficients, it can range from −1 to +1, where 0 represents the amount of agreement that can be expected from random chance, and 1 represents perfect agreement between the raters.

Calculation of Cohen’s kappa may be performed according to the following formula:

| 𝜅=Pr (𝑎)−Pr(𝑒) / 1−Pr(𝑒) |
| --- |

Where Pr(a) represents the actual observed agreement, and Pr(e) represents chance agreement.
