# Supplementary material for: Comparison of verbal autopsy using a large language model to biologically confirmed causes of death for malaria and other communicable diseases among children in six sub-Saharan African countries
Source: Malar J. 2026 Jan 6;25:77. doi: 10.1186/s12936-025-05774-z (PMC12870146; doi:10.1186/s12936-025-05774-z)
Supplement: Supplementary file 2 — Supplementary Material 2: Annex 3: Reclassification tables by communicable disease—MITS vs GPT-4o AI model. [file 12936_2025_5774_MOESM2_ESM.docx]

RECLASSIFICATION TABLES BY DISEASE CATEGORIES (MITS vs VA GPT-4o AI MODEL)

COMMUNICABLE DISEASES

Table 1

| **Level of reclassification of MITS Malaria-attributed deaths by GPT-4o AI model** | | | |
| --- | --- | --- | --- |
| **MITS Underlying CoD Malaria** | **GPT-4o Underlying CoD** | **No.** | **%** |
| Malaria (130) | Malaria (B50,B54) | 60 | 46 |
|  | Disseminated infections | 21 | 16 |
|  | Diarrhoeal diseases (A06, A09) | 8 | 6 |
|  | HIV/AIDS (B20, B23, B24) | 6 | 5 |
|  | Pneumonia (J18, P23) | 7 | 5 |
|  | Meningitis (G00, G01, G03, ) | 3 | 2 |
|  | Tuberculosis (A15, A19) | 2 | 2 |
|  | Other infections (A35, B05, P37, P39, A87, A90, B37, R50) | 11 | 8 |
|  | Diabetes Mellitus (E14,) | 2 | 2 |
|  | Other non-communicable diseases (E15, P60, E72, J90, P78) | 6 | 5 |
|  | Epilepsy (G40) | 2 | 2 |
|  | Other (P61) | 1 | 1 |
|  | Ill-defined | 1 | 1 |
|  | Total | 130 | 100 |

46% (60/130) of malaria-attributed deaths were correctly classified as malaria by GPT-4o in comparison to MITS. 16% were misclassified as disseminated infections/sepsis, while diarrhoeal diseases, HIV and pneumonia were misclassified 6%, 5% and 5% of the time, respectively.

Table 2

| **Level of reclassification of MITS Malaria-attributed deaths by GPT-4o AI model** | | | |
| --- | --- | --- | --- |
| **MITS Underlying CoD Malaria** | **GPT-4o Underlying CoD** | **No.** | **%** |
| Malaria (130) | Malaria (B50, B54) | 60 | 46 |
|  | Disseminated infections | 21 | 16 |
|  | Diarrhoeal diseases (A06, A09) | 8 | 6 |
|  | HIV/AIDS (B20, B23, B24) | 6 | 5 |
|  | Pneumonia (J18, P23) | 7 | 5 |
|  | Meningitis (G00, G01, G03, ) | 3 | 2 |
|  | Tuberculosis (A15, A19) | 2 | 2 |
|  | Other infections (A35, B05, P37, P39, A87, A90, B37, R50) | 11 | 8 |
|  | Other non-communicable diseases (E,14, E15, G40, P60, E72, J90, P78) | 10 | 8 |
|  | Other /Ill-defined (P61, R99) | 2 | 2 |
|  | Total | 130 | 100 |

Table 3a

| **MITS Underlying CoD for Non-Malaria Infectious Disease Records** | | | |
| --- | --- | --- | --- |
| **MITS Underlying CoD** | **ICD-10 codes** | **No.** | **%** |
| Pneumonia | A37, J05, J10, J12, J13, J14, J15, J16, J17, J18, J86, P23 | 86 | 29 |
| HIV/AIDS | B20, B22, B24 | 81 | 27 |
| Diarrhoeal disease | A00 - A09 | 60 | 20 |
| Disseminated infections | A39, A40, A41 | 39 | 13 |
| Other infections | A48, A50, A82, B05, B25, B33, B34, B89, B96, I41, K65, L01, L02, P35 | 21 | 7 |
| Tuberculosis | A15 - A19 | 6 | 2 |
| Meningitis/ Encephalitis | G00, G04 | 6 | 2 |
| Total |  | 299 | 100 |

Table 3b

| **MITS Underlying CoD for Communicable disease records** | | | |
| --- | --- | --- | --- |
| **MITS Underlying CoD** | **ICD-10** | **No.** | **%** |
| Malaria | B50 | 130 | 30 |
| Pneumonia | A37, J05, J10, J12, J13, J14, J15, J16, J17, J18, J86, P23 | 86 | 20 |
| HIV/AIDS | B20, B22, B24 | 81 | 19 |
| Diarrhoeal disease | A00 - A09 | 60 | 14 |
| Disseminated infections | A39, A40, A41 | 39 | 9 |
| Other infections | A48, A50, A82, B05, B25, B33, B34, B89, B96, I41, K65, L01, L02, P35 | 21 | 5 |
| Tuberculosis | A15 - A19 | 6 | 1 |
| Meningitis/ Encephalitis | G00, G04 | 6 | 1 |
| Total |  | 429 | 100 |

Table 4

| **Level of reclassification of MITS Pneumonia deaths by GPT-4o AI model** | | | | |
| --- | --- | --- | --- | --- |
| **MITS Underlying CoD Pneumonia** | **GPT-4o Underlying CoD** | **ICD-10 codes** | **No.** | **%** |
| Pneumonia (86) | Pneumonia | J18, P23 | 15 | 17 |
|  | Malaria | B50, B54 | 17 | 20 |
|  | Diarrhoeal diseases | A06, A09 | 12 | 14 |
|  | Other / Ill-defined | P22, P24, R68, R99 | 9 | 10 |
|  | HIV/AIDS | B20, B23, B24 | 8 | 9 |
|  | Disseminated infections | A41 | 8 | 9 |
|  | Other infections | A33, A35, B08, B37, P35, P36, P37 | 8 | 9 |
|  | other non-communicable dis. | E86, P56, P92, R56 | 4 | 5 |
|  | Malnutrition | E40,E42, E43 | 3 | 3 |
|  | Tuberculosis | A16 | 1 | 1 |
|  | Meningitis | G00 | 1 | 1 |
|  |  |  | 86 | 100 |

Only 17% (15/86) of the pneumonia-attributed deaths by MITS were correctly classified by GPT-4o. 20% of the pneumonia-attributed deaths due to MITS were misclassified as malaria, and 14% were misclassified as diarrhoeal diseases.

Table 5

| **Level of reclassification of MITS HIV/AIDS deaths by GPT-4o AI model** | | | | |
| --- | --- | --- | --- | --- |
| **MITS Underlying CoD HIV/AIDS** | **GPT-4o Underlying CoD** | **ICD-10 codes** | **No.** | **%** |
| HIV/AIDS (81) | HIV/AIDS | B20, B23, B24 | 45 | 56 |
|  | Malaria | B50, B54 | 10 | 12 |
|  | Diarrhoeal diseases | A00, A02, A06, A09 | 9 | 11 |
|  | Disseminated infections | A41 | 5 | 6 |
|  | Pneumonia | J18, P23 | 4 | 5 |
|  | Other infections | A33, P35, P37 | 4 | 5 |
|  | Malnutrition | E40, E43 | 3 | 4 |
|  | Other non-communicable dis. | E14 | 1 | 1 |
|  | Total |  | 81 | 100 |

HIV/AIDS deaths were correctly classified by GPT-4o 56% (45/81) of the time compared to MITS. Only 12% (10/81) were misclassified as malaria and 11% (9/81) as diarrhoeal disease deaths.

Table 6

| **Level of reclassification of MITS Diarrhoeal disease deaths by GPT-4o AI model** | | | | |
| --- | --- | --- | --- | --- |
| **MITS Underlying CoD Diarrhoeal dis.** | **GPT-4o Underlying CoD** | **ICD-10 codes** | **No.** | **%** |
| Diarrhoeal diseases (60) | Diarrhoeal Diseases | A06, A09 | 34 | 57 |
|  | Disseminated infections | A41 | 6 | 10 |
|  | HIV / AIDS | B20, B24 | 5 | 8 |
|  | Other infections | A35, B05, P36 | 4 | 7 |
|  | Other | P22, P28, P95 | 4 | 7 |
|  | Malaria | B50 | 3 | 5 |
|  | Pneumonia | J18, P23 | 2 | 3 |
|  | Other non-communicable dis. | L25, P92 | 2 | 3 |
|  | Total |  | 60 | 100 |

57% of diarrhoeal disease deaths were correctly classified as diarrhoea deaths by GPT-4o when compared to MITS. Diarrhoeal disease deaths were misclassified by GPT-4o by 10% and 8% of the time as disseminated infections and HIV deaths, respectively.

Table 7

| **Level of reclassification of MITS Disseminated infection deaths by GPT-4o AI model** | | | | |
| --- | --- | --- | --- | --- |
| **MITS Underlying CoD Disseminated infections** | **GPT-4o Underlying CoD** | **ICD-10 codes** | **No.** | **%** |
| Disseminated infections (39) | Disseminated infections | A41 | 6 | 15 |
|  | Malaria | B50, B54 | 8 | 21 |
|  | Diarrhoeal diseases | A06, A09 | 5 | 13 |
|  | Other / Ill-defined | P22, P61, R51, R99 | 5 | 13 |
|  | HIV /AIDS | B20 | 4 | 10 |
|  | Tuberculosis | A15, A16 | 3 | 8 |
|  | Pneumonia | J18, P23 | 3 | 8 |
|  | Other infections | A27, P37 | 3 | 8 |
|  | Other non-communicable dis | P92 | 1 | 3 |
|  | Congenital Anomaly | P21 | 1 | 3 |
|  | Total |  | 39 | 100 |

Only 15% (6/39) of disseminated infection deaths were classified correctly by GPT-4o. Deaths were misclassified 21% of the time as malaria and 13% for diarrhoeal disease deaths.

Table 8

| **Level of reclassification of MITS Other infection deaths by GPT-4o AI model** | | | | |
| --- | --- | --- | --- | --- |
| **MITS Underlying CoD Other infections** | **GPT-4o Underlying CoD** | **ICD-10 codes** | **No.** | **%** |
| Other Infections (21) | Other infections | A35, B05, P36, P37 | 5 | 24 |
|  | HIV /AIDS | B20 | 3 | 14 |
|  | Pneumonia | J18, P23 | 3 | 14 |
|  | Disseminated infections | A41 | 3 | 14 |
|  | Diarrhoeal diseases | A09 | 2 | 10 |
|  | Other /Ill-defined | R99 | 2 | 10 |
|  | Tuberculosis | A15 | 1 | 5 |
|  | Malaria | B50 | 1 | 5 |
|  | Other non-communicable dis | P83 | 1 | 5 |
|  | Total |  | 21 | 100 |

Deaths from other infections were correctly classified by GPT-4o 24% of the time.

Table 9

| **Level of reclassification of MITS Tuberculosis deaths by GPT-4o AI model** | | | | |
| --- | --- | --- | --- | --- |
| **MITS Underlying CoD Tuberculosis** | **GPT-4o Underlying CoD** | **ICD-10 codes** | **No.** | **%** |
| Tuberculosis (6) | Tuberculosis | A16 | 1 | 17 |
|  | Malaria | B50 | 2 | 33 |
|  | Diarrhoeal diseases | A06 | 1 | 17 |
|  | Pneumonia | P36 | 1 | 17 |
|  | Other infections | A27 | 1 | 17 |
|  | Total |  | 6 | 100 |

Table 10

| **Level of reclassification of MITS Meningitis/Encephalitis deaths by GPT-4o AI model** | | | | |
| --- | --- | --- | --- | --- |
| **MITS Underlying CoD Tuberculosis** | **GPT-4o Underlying CoD** | **ICD-10 codes** | **No.** | **%** |
| Meningitis / Encephalitis (6) | Meningitis /Encephalitis | G04 | 1 | 17 |
|  | Disseminated Infections | A41 | 2 | 33 |
|  | Pneumonia | J18 | 1 | 17 |
|  | Other infections | A90 | 1 | 17 |
|  | Ill-defined | R51 | 1 | 17 |
|  | Total |  | 6 | 100 |

Table 10
