# Supplementary material for: Comparison of verbal autopsy using a large language model to biologically confirmed causes of death for malaria and other communicable diseases among children in six sub-Saharan African countries
Source: Malar J. 2026 Jan 6;25:77. doi: 10.1186/s12936-025-05774-z (PMC12870146; doi:10.1186/s12936-025-05774-z)
Supplement: Supplementary file 3 — Supplementary Material 3: Annex 4: Alluvial diagram showing Communicable disease CoD differences by MITS and InSilicoVA. [file 12936_2025_5774_MOESM3_ESM.docx]

**ANNEX 4**


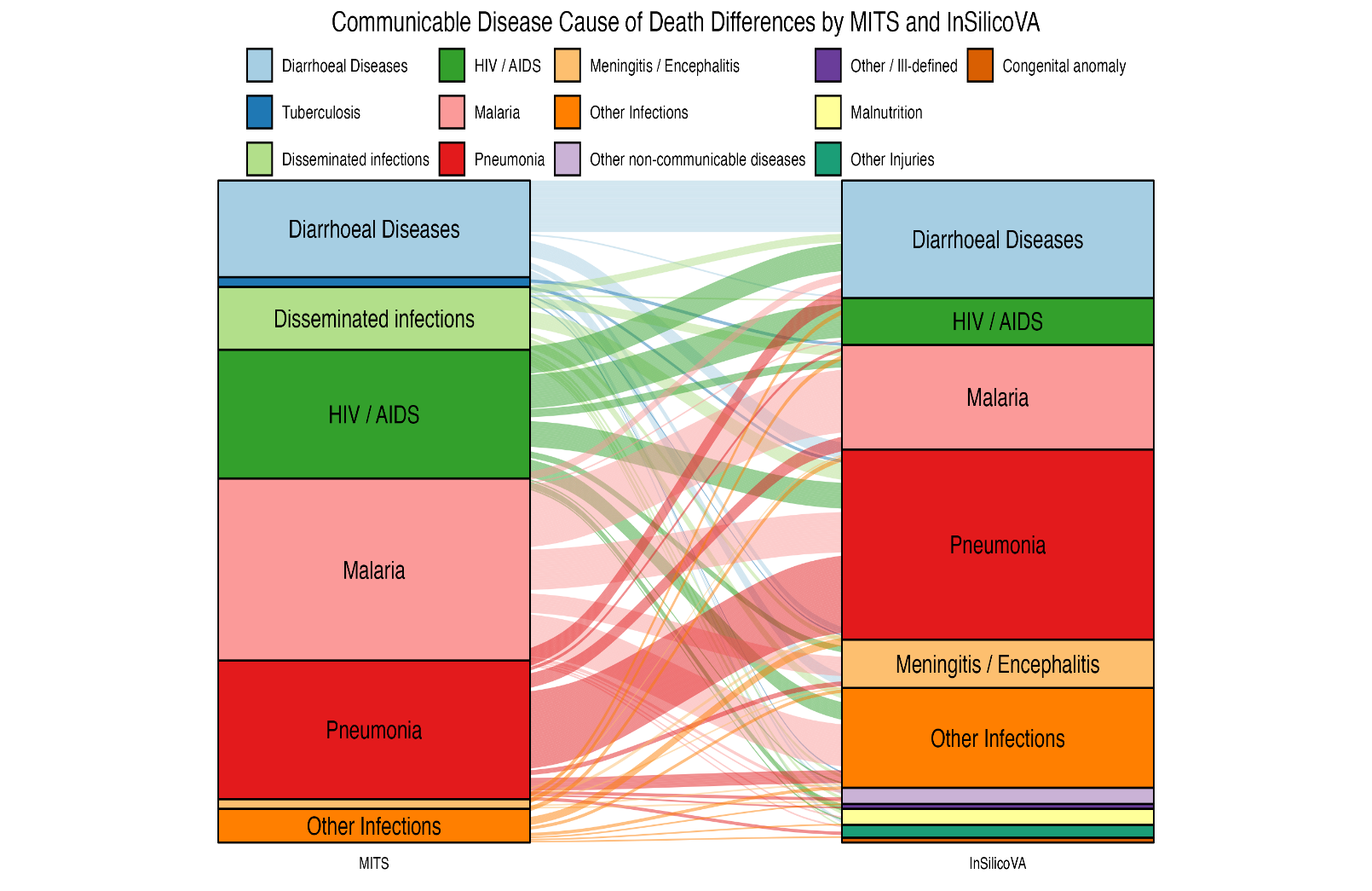


Figure: Alluvial diagram showing Communicable disease CoD differences by MITS and InSilicoVA
