# Supplementary material for: Comparison of verbal autopsy using a large language model to biologically confirmed causes of death for malaria and other communicable diseases among children in six sub-Saharan African countries
Source: Malar J. 2026 Jan 6;25:77. doi: 10.1186/s12936-025-05774-z (PMC12870146; doi:10.1186/s12936-025-05774-z)
Supplement: Supplementary file 4 — Supplementary Material 4: Annex 5: Reclassification tables by communicable disease—MITS vs InSilicoVA model. [file 12936_2025_5774_MOESM4_ESM.docx]

**RECLASSIFICATION TABLES BY DISEASE CATEGORIES (MITS vs InSilicoVA MODEL)**

**COMMUNICABLE DISEASES**

Table 1

| **MITS Underlying CoD for Communicable disease records** | | | |
| --- | --- | --- | --- |
| **MITS Underlying CoD** | **ICD-10** | **No.** | **%** |
| Malaria | B50 | 130 | 30 |
| Pneumonia | A37, J05, J10, J12, J13, J14, J15, J16, J17, J18, J86, P23 | 86 | 20 |
| HIV/AIDS | B20, B22, B24 | 81 | 19 |
| Diarrhoeal disease | A00 - A09 | 60 | 14 |
| Disseminated infections | A39, A40, A41 | 39 | 9 |
| Other infections | A48, A50, A82, B05, B25, B33, B34, B89, B96, I41, K65, L01, L02, P35 | 21 | 5 |
| Tuberculosis | A15 - A19 | 6 | 1 |
| Meningitis/ Encephalitis | G00, G04 | 6 | 1 |
| Total |  | 429 | 100 |

Table 2

| **Level of reclassification of MITS Malaria deaths by the InSilicoVA model** | | | | |
| --- | --- | --- | --- | --- |
| **MITS Underlying CoD Malaria** | **InSilicoVA Underlying CoD** | **ICD-10 codes** | **No.** | **%** |
| Malaria (130) | Malaria | B54 | 39 | 30 |
|  | Other infections | B99 | 26 | 20 |
|  | Pneumonia | J22 | 25 | 19 |
|  | Other / Ill-defined | R10, R99 | 18 | 14 |
|  | Meningitis | G03 | 12 | 9 |
|  | Diarrhoeal diseases | A09 | 5 | 4 |
|  | other non-communicable dis. | E14, I64 | 2 | 2 |
|  | HIV/AIDS | B24 | 1 | 1 |
|  | Malnutrition | E46 | 1 | 1 |
|  | Other Injuries | V89 | 1 | 1 |
| Total |  |  | 130 | 100 |

30% (39/130) of malaria deaths were correctly classified as malaria by InSilicoVA in comparison to MITS. 20% (26/130) were misclassified as other infections, while pneumonia, other/ill-defined, and meningitis were misclassified at 19%, 14%, and 9%, respectively.

Table 3

| **Level of reclassification of MITS Pneumonia deaths by InSilicoVA model** | | | | |
| --- | --- | --- | --- | --- |
| **MITS Underlying CoD Pneumonia** | **InSilicoVA Underlying CoD** | **ICD-10 codes** | **No.** | **%** |
| Pneumonia (86) | Pneumonia | J22, A37 | 48 | 56 |
|  | Diarrhoeal diseases | A09 | 11 | 13 |
|  | Malaria | B54 | 9 | 10 |
|  | Other infections | A99, B99 | 8 | 9 |
|  | Meningitis | G03 | 3 | 3 |
|  | HIV/AIDS | B24 | 2 | 2 |
|  | other non-communicable dis. | D57, I24 | 2 | 2 |
|  | Other Injuries | V99, W19 | 2 | 2 |
|  | Other / Ill-defined | R10 | 1 | 1 |
| Total |  |  | 86 | 100 |

56% (48/86) of pneumonia-attributed deaths by MITS were correctly classified by InSilicoVA. 13% and 10% of the deaths were misclassified as diarrhoeal diseases and malaria respectively.

Table 4

| **Level of reclassification of MITS HIV/AIDS deaths by InSilicoVA model** | | | | |
| --- | --- | --- | --- | --- |
| **MITS Underlying CoD HIV/AIDS** | **InSilicoVA Underlying CoD** | **ICD-10 codes** | **No.** | **%** |
| HIV/AIDS (81) | HIV/AIDS | B24 | 21 | 26 |
|  | Malaria | B54 | 5 | 6 |
|  | Diarrhoeal diseases | A09 | 17 | 21 |
|  | Meningitis | G03 | 4 | 5 |
|  | Pneumonia | J22 | 16 | 20 |
|  | Other infections | B99 | 11 | 14 |
|  | Malnutrition | E46 | 4 | 5 |
|  | Other non-communicable dis. | N19 | 1 | 1 |
|  | Other / Ill-defined | R99 | 1 | 1 |
|  | Other Injuries | V89 | 1 | 1 |
| Total |  |  | 81 | 100 |

26% (21/81) of HIV attributed deaths by MITS were correctly classified by InSilicoVA. 21% and 20% of the deaths were misclassified as diarrhoeal diseases and pneumonia respectively.

Table 5

| **Level of reclassification of MITS Diarrhoeal disease deaths by InSilicoVA model** | | | | |
| --- | --- | --- | --- | --- |
| **MITS Underlying CoD Diarrhoeal dis.** | **InSilicoVA Underlying CoD** | **ICD-10 codes** | **No.** | **%** |
| Diarrhoeal diseases (60) | Diarrhoeal Diseases | A09 | 32 | 53 |
|  | Pneumonia | J22 | 10 | 17 |
|  | Other infections | A90, B99 | 8 | 13 |
|  | Meningitis | G03 | 4 | 7 |
|  | Malnutrition | E46 | 2 | 3 |
|  | Congenital Anomalies | P07, P21 | 2 | 3 |
|  | HIV / AIDS | B24 | 1 | 2 |
|  | Other | P95 | 1 | 2 |
|  | Total |  | 60 | 100 |

53 % (32/60) of diarrhoeal disease-attributed deaths by MITS were correctly classified as diarrhoeal disease. 17% and 13% were misclassified as pneumonia and other infections, respectively.

Table 6

| **Level of reclassification of MITS Disseminated infection deaths by InSilicoVA model** | | | | |
| --- | --- | --- | --- | --- |
| **MITS Underlying CoD Disseminated infections** | **InSilicoVA Underlying CoD** | **ICD-10 codes** | **No.** | **%** |
| Disseminated infections (39) | Malnutrition | E46 | 2 | 5 |
|  | Malaria | B54 | 6 | 15 |
|  | Diarrhoeal diseases | A09 | 5 | 13 |
|  | Other Injuries | V89, W19 | 3 | 8 |
|  | HIV /AIDS | B24 | 1 | 3 |
|  | Malnutrition | E46 | 3 | 8 |
|  | Pneumonia | J22 | 10 | 26 |
|  | Other infections | B99 | 6 | 15 |
|  | Other non-communicable dis | G40 | 2 | 5 |
|  | Congenital Anomaly | Q89 | 1 | 3 |
|  | Total |  | 39 | 100 |

Deaths due to disseminated infections by MITS were misclassified mostly as pneumonia (26%), malaria (15%) and other infections (15%) by InSilicoVA.

Table 7

| **Level of reclassification of MITS Other infection deaths by InSilicoVA model** | | | | |
| --- | --- | --- | --- | --- |
| **MITS Underlying CoD Other infections** | **InSilicoVA Underlying CoD** | **ICD-10 codes** | **No.** | **%** |
| Other Infections (21) | Pneumonia | J22 | 5 | 24 |
|  | Malaria | B54 | 3 | 14 |
|  | HIV /AIDS | B24 | 3 | 14 |
|  | Diarrhoeal diseases | A09 | 3 | 14 |
|  | Other infections | B05, B99 | 2 | 10 |
|  | Meningitis | G03 | 2 | 10 |
|  | Other non-communicable dis | I64 | 1 | 5 |
|  | Other Injuries | W19 | 1 | 5 |
|  | Malnutrition | E46 | 1 | 5 |
|  | Total |  | 21 | 100 |

Table 8

| **Level of reclassification of MITS Tuberculosis deaths by InSilicoVA model** | | | | |
| --- | --- | --- | --- | --- |
| **MITS Underlying CoD Tuberculosis** | **InSilicoVA Underlying CoD** | **ICD-10 codes** | **No.** | **%** |
| Tuberculosis (6) | Meningitis | G03 | 1 | 17 |
|  | Malaria | B54 | 2 | 33 |
|  | Pneumonia | J22 | 2 | 33 |
|  | Other Non-Communicable dis | I64 | 1 | 17 |
|  | Total |  | 6 | 100 |

Table 9

| **Level of reclassification of MITS Meningitis/Encephalitis deaths by InSilicoVA model** | | | | |
| --- | --- | --- | --- | --- |
| **MITS Underlying CoD Meningitis / Encephalitis** | **InSilicoVA Underlying CoD** | **ICD-10 codes** | **No.** | **%** |
| Meningitis / Encephalitis (6) | Meningitis /Encephalitis | G03 | 1 | 17 |
|  | Malaria | B54 | 1 | 17 |
|  | Pneumonia | J22 | 2 | 33 |
|  | Other infections | B99 | 1 | 17 |
|  | Other Non-Communicable dis | G40 | 1 | 17 |
|  | Total |  | 6 | 100 |

Table 10
