# Supplementary material for: Comparison of verbal autopsy using a large language model to biologically confirmed causes of death for malaria and other communicable diseases among children in six sub-Saharan African countries
Source: Malar J. 2026 Jan 6;25:77. doi: 10.1186/s12936-025-05774-z (PMC12870146; doi:10.1186/s12936-025-05774-z)
Supplement: Supplementary file 6 — Supplementary Material 6: Annex 7: Reclassification tables by communicable disease—MITS vs InterVA-5 model. [file 12936_2025_5774_MOESM6_ESM.docx]

**RECLASSIFICATION TABLES BY DISEASE CATEGORIES (MITS vs InterVA-5 MODEL)**

**COMMUNICABLE DISEASES**

Table 1

| **MITS Underlying CoD for Communicable disease records** | | | |
| --- | --- | --- | --- |
| **MITS Underlying CoD** | **ICD-10** | **No.** | **%** |
| Malaria | B50 | 130 | 30 |
| Pneumonia | A37, J05, J10, J12, J13, J14, J15, J16, J17, J18, J86, P23 | 86 | 20 |
| HIV/AIDS | B20, B22, B24 | 81 | 19 |
| Diarrhoeal disease | A00 - A09 | 60 | 14 |
| Disseminated infections | A39, A40, A41 | 39 | 9 |
| Other infections | A48, A50, A82, B05, B25, B33, B34, B89, B96, I41, K65, L01, L02, P35 | 21 | 5 |
| Tuberculosis | A15 - A19 | 6 | 1 |
| Meningitis/ Encephalitis | G00, G04 | 6 | 1 |
| Total |  | 429 | 100 |

Table 2

| **Level of reclassification of MITS Malaria deaths by the InterVA-5 model** | | | | |
| --- | --- | --- | --- | --- |
| **MITS Underlying CoD Malaria** | **InterVA-5 Underlying CoD** | **ICD-10 codes** | **No.** | **%** |
| Malaria (130) | Malaria | B54 | 30 | 23 |
|  | Other infections | B99 | 15 | 12 |
|  | Pneumonia | J22 | 18 | 14 |
|  | Other / Ill-defined | R10, R99 | 25 | 19 |
|  | Meningitis | G03 | 18 | 14 |
|  | Diarrhoeal diseases | A09 | 12 | 9 |
|  | other non-communicable dis. | E14, I24, I64, I99, K74 | 6 | 5 |
|  | HIV/AIDS | B24 | 1 | 1 |
|  | Malnutrition | E46 | 1 | 1 |
|  | Disseminated Infections | A41 | 1 | 1 |
|  | Congenital Anomaly | Q89 | 2 | 2 |
|  | Other Injuries | X59 | 1 | 1 |
| Total |  |  | 130 | 100 |
| 23% (30/130) of the malaria-attributed deaths were correctly classified by InterVA-5. Many deaths were misclassified by InterVA-5 as Other/Ill-defined (19%), Meningitis (14%), Pneumonia (14%) and other infections (12%) according to MITS. | | | | |
| Table 3 |  |  |  |  |
| **Level of reclassification of MITS Pneumonia deaths by InterVA-5 model** | | | | |
| **MITS Underlying CoD Pneumonia** | **InterVA-5 Underlying CoD** | **ICD-10 codes** | **No.** | **%** |
| Pneumonia (86) | Pneumonia | J22, | 20 | 23 |
|  | Diarrhoeal diseases | A09 | 17 | 20 |
|  | Malaria | B54 | 5 | 6 |
|  | Other infections | A35, B99 | 12 | 14 |
|  | Meningitis | G03 | 8 | 9 |
|  | HIV/AIDS | B24 | 1 | 1 |
|  | other non-communicable dis. | G40, I24, I99, K74, N19 | 6 | 7 |
|  | Other Injuries | W19 | 1 | 1 |
|  | Malnutrition | E46 | 4 | 5 |
|  | Disseminated Infections | A41 | 1 | 1 |
|  | Congenital Anomaly | Q89 | 3 | 3 |
|  | Other / Ill-defined | R10, R99 | 8 | 9 |
| Total |  |  | 86 | 100 |
| Pneumonia was correctly classified in 23% (20/86) of the deaths due to MITS. Many deaths were misclassified as diarrhoeal diseases (20%) and other infections (14%). | | | | |
| Table 4 |  |  |  |  |
| **Level of reclassification of MITS HIV/AIDS deaths by InterVA-5 model** | | | | |
| **MITS Underlying CoD HIV/AIDS** | **InterVA-5 Underlying CoD** | **ICD-10 codes** | **No.** | **%** |
| HIV/AIDS (81) | HIV/AIDS | B24 | 20 | 25 |
|  | Malaria | B54 | 2 | 2 |
|  | Diarrhoeal diseases | A09 | 20 | 25 |
|  | Meningitis | G03 | 3 | 4 |
|  | Pneumonia | J22 | 6 | 7 |
|  | Other infections | B99 | 10 | 12 |
|  | Malnutrition | E46 | 4 | 5 |
|  | Other non-communicable dis. | D57, G40, I24, I64, I99, N19 | 7 | 9 |
|  | Other / Ill-defined | R10, R99 | 9 | 11 |
| Total |  |  | 81 | 100 |
| 25% (20/81) of the deaths due to HIV/AIDS were correctly classified by InterVA-5 according to MITS. Many deaths were misclassified as diarrhoeal diseases (25%) and other infections (12%). | | | | |
| Table 5 |  |  |  |  |
| **Level of reclassification of MITS Diarrhoeal disease deaths by InterVA-5 model** | | | | |
| **MITS Underlying CoD Diarrhoeal dis.** | **InterVA-5 Underlying CoD** | **ICD-10 codes** | **No.** | **%** |
| Diarrhoeal diseases (60) | Diarrhoeal Diseases | A09 | 31 | 52 |
|  | Pneumonia | J22 | 3 | 5 |
|  | Other infections | B99 | 6 | 10 |
|  | Meningitis | G03 | 5 | 8 |
|  | Congenital Anomalies | P07, Q89 | 4 | 7 |
|  | HIV / AIDS | B24 | 1 | 2 |
|  | Other / Ill-defined | P95, R99 | 10 | 17 |
|  | Total |  | 60 | 100 |
| 52% of deaths due to diarrhoeal diseases were correctly classified by InterVA-5 according to MITS. | | | | |
| Table 6 |  |  |  |  |
| **Level of reclassification of MITS Disseminated infection deaths by InterVA-5 model** | | | | |
| **MITS Underlying CoD Disseminated infections** | **InterVA-5 Underlying CoD** | **ICD-10 codes** | **No.** | **%** |
| Disseminated infections (39) | Disseminated Infections | A41 | 1 | 3 |
|  | Malaria | B54 | 2 | 5 |
|  | Diarrhoeal diseases | A09 | 9 | 23 |
|  | Other Injuries | W19 | 1 | 3 |
|  | Ill-defined | R99 | 7 | 18 |
|  | Meningitis | G03 | 3 | 8 |
|  | Pneumonia | J22 | 5 | 13 |
|  | Other infections | A35, B99 | 7 | 18 |
|  | Other non-communicable dis | G40 | 3 | 8 |
|  | Congenital Anomaly | Q89 | 1 | 3 |
|  | Total |  | 39 | 100 |
| Deaths due to Disseminated infections were mostly classified as diarrhoeal diseases (23%), other infections (18%) and pneumonia (13%). | | | | |
| Table 7 |  |  |  |  |
| **Level of reclassification of MITS Other infection deaths by InterVA-5 model** | | | | |
| **MITS Underlying CoD Other infections** | **InterVA-5 Underlying CoD** | **ICD-10 codes** | **No.** | **%** |
| Other Infections (21) | Pneumonia | J22 | 3 | 14 |
|  | Malaria | B54 | 2 | 10 |
|  | HIV /AIDS | B24 | 1 | 5 |
|  | Diarrhoeal diseases | A09 | 2 | 10 |
|  | Other infections | B05, B99 | 3 | 14 |
|  | Meningitis | G03 | 2 | 10 |
|  | Other non-communicable dis | E14, K74, N19, I64 | 4 | 19 |
|  | Other Injuries | W19 | 1 | 5 |
|  | Other /Ill-defined | R99 | 2 | 10 |
|  | Malnutrition | E46 | 1 | 5 |
|  | Total |  | 21 | 100 |
| Table 8 |  |  |  |  |
| **Level of reclassification of MITS Tuberculosis deaths by InterVA-5 model** | | | | |
| **MITS Underlying CoD Tuberculosis** | **InterVA-5 Underlying CoD** | **ICD-10 codes** | **No.** | **%** |
| Tuberculosis (6) | Meningitis | G03 | 1 | 17 |
|  | Malaria | B54 | 1 | 17 |
|  | Pneumonia | J22 | 2 | 33 |
|  | HIV /AIDS | B24 | 1 | 17 |
|  | Other Non-Communicable dis | I64 | 1 | 17 |
|  | Total |  | 6 | 100 |
| Table 9 |  |  |  |  |
| **Level of reclassification of MITS Meningitis/Encephalitis deaths by InterVA-5 model** | | | | |
| **MITS Underlying CoD Meningitis / Encephalitis** | **InterVA-5 Underlying CoD** | **ICD-10 codes** | **No.** | **%** |
| Meningitis / Encephalitis (6) | Meningitis /Encephalitis | G03 | 1 | 17 |
|  | Other /Ill-defined | R99 | 1 | 17 |
|  | Pneumonia | J22 | 1 | 17 |
|  | Other infections | B99 | 1 | 17 |
|  | Other Non-Communicable dis | G40 | 2 | 33 |
|  | Total |  | 6 | 100 |

Table 10
